# TSH suppression aggravates arterial inflammation — an ^18^F-FDG PET study in thyroid carcinoma patients

**DOI:** 10.1007/s00259-019-04292-w

**Published:** 2019-03-11

**Authors:** Ellen Boswijk, Karin J. C. Sanders, Evie P. M. Broeders, Marlies de Ligt, Guy H. E. J. Vijgen, Bas Havekes, Alma M. A. Mingels, Roel Wierts, Wouter D. van Marken Lichtenbelt, Patrick Schrauwen, Felix M. Mottaghy, Joachim E. Wildberger, Jan Bucerius

**Affiliations:** 1 Department of Radiology and Nuclear Medicine, Maastricht University Medical Centre (MUMC+), P. Debyelaan 25, 6229 HX Maastricht, The Netherlands; 2 Cardiovascular Research Institute Maastricht (CARIM), Maastricht University, Universiteitssingel 50, 6229 ER Maastricht, The Netherlands; 3 Department of Nutrition and Movement Sciences, School of Nutrition and Translational Research in Metabolism (NUTRIM), Maastricht University, Universiteitssingel 40, 6229 ER Maastricht, The Netherlands; 4 Department of Respiratory Medicine, School of Nutrition and Translational Research in Metabolism (NUTRIM), Universiteitssingel 40, 6229 ER Maastricht, The Netherlands; 5 Department of Family Medicine, Amsterdam University Medical Centre (Amsterdam UMC), Meibergdreef 9, 1105 AZ Amsterdam, the Netherlands; 6 Department of Surgery, Franciscus, Kleiweg 500, 3045 PM Rotterdam, The Netherlands; 7 Department of Internal Medicine, Division of Endocrinology, Maastricht University Medical Centre (MUMC+), P. Debyelaan 25, 6229 HX Maastricht, The Netherlands; 8 Department of Clinical Chemistry, Central Diagnostic Laboratory, Maastricht University Medical Centre (MUMC+), P. Debyelaan 25, 6229 HX Maastricht, The Netherlands; 9 Department of Nuclear Medicine, University Hospital RWTH Aachen, Pauwelsstraße 30, 52074 Aachen, Germany

**Keywords:** Positron emission tomography (PET), Thyroid disease, Atherosclerosis, Inflammation, TSH suppression

## Abstract

**Purpose:**

We aimed to investigate the influence of both hypothyroidism and thyroid-stimulating hormone (TSH) suppression on vascular inflammation, as assessed with ^18^F-fluorodeoxyglucose (^18^F-FDG) positron emission tomography (PET)/computed tomography (CT).

**Methods:**

Ten thyroid carcinoma patients underwent an ^18^F-FDG PET/CT during post-thyroidectomy hypothyroidism and during thyrotropin (TSH) suppression after ^131^I (radioiodine) ablation therapy. We analysed the ^18^F-FDG uptake in the carotids, aortic arch, ascending, descending, and abdominal aorta to investigate the effects of thyroid hormone status on arterial inflammation. Target-to-background ratios (TBRs) corrected for blood pool activity were established for all arterial territories. Results were further compared to euthyroid historic control subjects.

**Results:**

In general, there was a trend towards higher vascular TBRs during TSH suppression than during hypothyroidism (TBR_max_ all vessels = 1.6 and 1.8, respectively, *p* = 0.058), suggesting a higher degree of arterial inflammation. In concurrence with this, we found increased C-reactive protein (CRP) levels after levothyroxine treatment (CRP = 2.9 mg/l and 4.8 mg/l, *p* = 0.005). An exploratory comparison with euthyroid controls showed significant higher TBRs during TSH suppression for the carotids, aortic arch, thoracic descending aorta, and when all vascular territories were combined (TBR_max_*p* = 0.013, *p* = 0.016, *p* = 0.030 and *p* = 0.018 respectively).

**Conclusions:**

Arterial inflammation is increased during TSH suppression. This finding sheds new light on the underlying mechanism of the suspected increased risk of cardiovascular disease in patients with TSH suppression.

**Electronic supplementary material:**

The online version of this article (10.1007/s00259-019-04292-w) contains supplementary material, which is available to authorized users.

## Background

In order to prevent cardiovascular events, it is necessary to identify subjects with a high risk of developing advanced atherosclerosis. Several clinical risk factors for atherosclerosis and subsequent complications have been identified, such as obesity, hypertension, diabetes, and smoking [1]. A less commonly known risk factor is thyroid hormone imbalance. Although findings for both hypo- and particularly hyperthyroidism have been ambiguous, it is suspected that both influence atherosclerosis negatively, resulting in a U-shaped correlation between thyroid hormone levels and the risk of cardiovascular disease [2]. Since inflammation plays a major role in cardiovascular disease, it could be expected that thyroid hormone status influences atherosclerosis via arterial inflammation.

A commonly used method to image arterial inflammation is ^18^F-fluorodeoxyglucose (^18^F-FDG) positron emission tomography (PET). In atherosclerosis, ^18^F-FDG uptake has been correlated to macrophage content of carotid plaques [3], but also to the presence or the development of symptoms [4, 5]. Additionally, the same technique has proven its value as a non-invasive method to measure the metabolic activity of other tissues, such as hematopoietic organs and subcutaneous and visceral adipose tissue (SAT and VAT) [6, 7]. Arterial inflammation has been correlated to an increased activation in the spleen and the bone marrow (BM) [8, 9] and to the metabolic activity in adipose tissue, most strongly in VAT [6, 7].

Patients with differentiated thyroid carcinoma (DTC) are commonly subjected to both ends of the thyroid hormone spectrum as a result of their therapy [10, 11]. After initial thyroidectomy, patients are left hypothyroid before adjuvant radioactive iodine (I^131^) ablation to facilitate uptake of radioactive iodine in the remaining thyroid tissue. Whereas thyroid hormone levels drop, thyrotropin [thyroid-stimulating hormone (TSH)] levels rise during this period. Since DTC cells express TSH receptors, high TSH levels will stimulate potential residual malignant tissue proliferation, but also iodine uptake, increasing the effect of I^131^ ablation [10, 11]. After therapy, patients are subscribed long-term levothyroxine treatment to induce a state of TSH suppression to prevent further stimulation of possible residual malignant tissue [10–12]. However, recent guidelines emphasize a personalized approach, weighing individual risk of recurrent disease against the risk of adverse effects of TSH suppression [10–13].

We aimed to investigate the influence of thyroid function on vascular inflammation, as assessed with ^18^F-FDG PET / computed tomography (CT). For this purpose, we analysed data from a prospective observational study on the effects of thyroid hormone on cold-induced brown adipose tissue (BAT) activation by ^18^F-FDG PET/CT in ten patients with DTC during both iatrogenic hypothyroidism and TSH suppression.

## Methods

### DTC subjects

Ten patients with DTC (papillary, *n* = 7; follicular, *n* = 3: two males and eight females) were included between June 2012 and July 2014. All patients were screened on their ability to comply with the study protocol, and all female patients had to be either postmenopausal or using a specific oral contraceptive pill to ensure stable sex hormone status. Exclusion criteria were insulin-dependent type 2 diabetes or diabetes-related complications, the use of β-blockers, pregnancy, recent participation in an intensive weight-loss program, and alcohol and/or drug abuse. The protocol was approved by the local medical ethics committee. All participants underwent two ^18^F-FDG PET/CT scans to study cold-induced BAT and thus served as their own control. Participants were treated for well-differentiated thyroid carcinoma according to local protocol and the national guideline for diagnosis and treatment of thyroid carcinoma [10]. This advocates thyroid resection, followed by a minimum period of 4 weeks without thyroid hormone replacement (withdrawal phase) to achieve the desired hypothyroidism. The first ^18^F-FDG PET/CT scan was performed when plasma fT4 levels were at a minimum (on average 6.8 ± 3.2 weeks after surgery, fT4 3.4 ± 0.8 pmol/l, TSH 104.9 ± 53.6 mU/l). During the same time, various measurements were performed to determine energy expenditure (see Broeders et al. [14] for details) and patients were scheduled for radioactive iodine ablation with ^131^I the same day. The second scan was carried out 4 to 6 months after radioactive iodine ablation therapy (22.7 ± 7.8 weeks), when patients had stable high fT4 and low TSH levels due to levothyroxine supplementation. Levothyroxine dosage had remained unchanged for at least a month before the second scan. The average dose was 143.8 ± 23.8 μg/day and during this period average fT4 and TSH levels were 23.1 ± 3.9 pmol/l and 0.5 ± 0.6 mU/l (range < 0.01–1.7) respectively.

### Euthyroid subjects

As euthyroid controls, we used data from placebo scans of eight participants of a crossover study evaluating the effect of resveratrol supplementation on BAT activation in subjects at risk of developing type 2 diabetes [15]. Participants had no known history of thyroid disease or medication that could interfere with thyroid hormones. These participants were included between April 2014 and March 2016. The protocol was approved by the local medical ethics committee. Participants were included primarily based on impaired insulin-sensitivity, defined as a glucose clearance rate of < 350 ml/kg/min according to the oral glucose insulin sensitivity (OGIS_120_) model, based on a standardized 2-h oral glucose tolerance test (OGTT). Furthermore, all participants were obese, had a sedentary life style, and had first-degree relatives with type 2 diabetes. Similarly to the study protocol of the DTC patients, all participants underwent two ^18^F-FDG PET/CT scan to study cold-induced BAT; once at the end of a 34-day placebo treatment period, and once at the end of a 34-day resveratrol treatment. Data from the placebo period were used for comparison to the DTC patients. Apart from the placebo treatment, all interventions, such as cold acclimatization protocol, were comparable between these studies. To confirm euthyroid state, fT4 and TSH levels were determined in preserved blood samples. Seven subjects were indeed euthyroid, but one subject had aTSH level in the high-normal range (4.23 mU/l) with a normal fT4. As a value of 4.0 mU/l is frequently considered as the upper limit of TSH normal range, we dicided a priorily to exclude this subject from further analysis to safely avoid any bias to the results.

### Laboratory tests

Fasting blood (EDTA plasma) was drawn on the day of either scan in the case of the DTC patients, and on day 30 of placebo treatment for the subjects at risk of developing type 2 diabetes. TSH, fT4, CRP, glucose, and cholesterol levels were analysed. TSH was measured using an electrochemiluminescent immunometric assay (Cobas 6000, Roche Diagnostics, Mannheim, Germany) with a reference range of 0.4–4.3 mU/l. fT4 was measured using a competitive immunofluorimetric assay (AutoDelfia, Perkin Elmer, Turku, Finland) with a reference range of 8–18 pmol/l. CRP was measured using a turbidimetric assay (Cobas 8000) with a reference range of < 10 mg/l and a detection limit of > 1 mg/l. Glucose was measured using a hexokinase-based assay (Cobas 6000) with a reference range of 3.1–7.8 mmol/l. Cholesterol levels were determined using spectrophotometric analysis (reference ranges total cholesterol < 5.0 mmol/l; LDL < 3.0 mmol/l). Plasma free fatty acids (FFA) and total glycerol were measured using enzymatic assays automated on a Cobas Fara/Mira analyser, using Wako Nefa C test kit (Wako Chemicals, Richmond, VA, USA) and Enzytec glycerol kit (R-biopharm, Darmstadt, Germany) respectively.

### PET protocol

All subjects underwent the same PET scan protocol which included an overnight fast, a dedicated and personalized cooling protocol, and the injection of a fixed activity of approximately 75 MBq ^18^F-FDG (details can be found in Broeders et al. [14] and de Ligt et al. [15]). Imaging was performed using a Gemini™ TF PET/CT system (Philips Healthcare, Eindhoven, The Netherlands). Patients underwent a low-dose non-contrast-enhanced whole-body CT protocol (120 kVp, 30 mAs) for scatter and attenuation correction and anatomical reference imaging.

### Image analysis

All image analyses were performed on a dedicated commercially available workstation (Extended Brilliance Workspace™ V4.5.3.40140, Philips). Circular regions of interest (ROIs) were manually drawn to encompass the entire artery including the arterial wall and lumen. The aorta was divided into ascending aorta, aortic arch, thoracic descending aorta (until the diaphragm) and abdominal aorta (until the iliac bifurcation or as low as sufficiently imaged).

For each ROI, the measured activity concentration was corrected for radioactive decay, total administered activity, and body weight, resulting in the mean and maximum standardized uptake value, SUV_mean_ and SUV_max_, respectively. To normalize for blood pool activity, a target-to-background ratio (TBR) was calculated by dividing the arterial SUVs by the mean SUV_mean_ of at least three standardized circular ROIs (4 mm) placed in the lumen of adjacent veins. The jugular vein (JV) served as a reference for the carotids and the superior cava vein (SCV) for the aorta. All measures for SUV_mean_ and SUV_max_ were corrected, which led to a corresponding TBR_mean_ and TBR_max_ respectively. The average of each vascular territory [whole vessel analysis (WVA)] was used for statistical analysis between the two different scans. For the carotid arteries, only the highest value was used for further analysis.

Standardized circular ROIs were also placed in bone marrow (20-mm ROIs placed in the centre of each thoracic and, if imaged, lumbar vertebra), spleen (20-mm ROIs encompassing all transversal slices), visceral (white) adipose tissue (VAT) (three 4-mm ROIs in intra-peritoneal fat around the level of the navel) and subcutaneous adipose tissue (SAT) in the neck (three 4-mm ROIs in the nuchal SAT) and chest (three 4-mm ROIs in SAT at the level of the xiphoid). TBRs were calculated by dividing corresponding SUVs by the mean SUV_mean_ of nearest reference vein (SCV in all cases except for nuchal SAT, where the JV was used).

In general, ROIs were excluded from further analysis, when spill-in of activity from adjacent structures with high uptake was suspected or motion-artefacts interfered with the drawing of representable ROIs.

### Statistical analysis

Statistical analysis was performed with IBM SPSS Statistics for Macintosh, version 23 (2015). Descriptive data are presented as mean values with the standard deviation (SD) or as absolute numbers. Because of the small group size, non-parametric tests were used. Continuous variables were compared between treatment periods using the Wilcoxon signed-rank test. The Mann–Whitney U test was used to compare continuous data between hypothyroidism and TSH suppression, with an independent sample of participants serving as a euthyroid controls. The χ^2^ test was used to compare categorical data between the different groups. Correlations between laboratory results and TBRs were tested using Spearman’s correlation coefficient. A *p* value of < 0.05 was considered to be statistically significant.

## Results

### Baseline characteristics and blood analysis

TSH and fT4 blood levels confirmed that all patients were hypothyroid at the time of the first scan (mean TSH 104.9 ± 53.6 mU/l; mean fT4 3.4 ± 0.8 pmol/l). During the second scan, all patients had had stable levothyroxine treatment (mean dose 143.8 ± 23.8 μg/day) for at least 1 month (mean TSH 0.5 ± 0.6 mU/l; fT4 23.1 ± 3.9 pmol/l). TSH was fully suppressed (< 0.01 mU/l) in 4/10 participants, suppressed (0.1 mU/l) in 2/10 and in the low-normal range (0.4–2 mU/l) in 4/10. In all but one patient, fT4 levels were above normal reference values (> 18 pmol/l) at the time of the second scan. One patient had an fT4 value in the high-normal range (14.3 pmol/l) corresponding to a TSH in the low-normal range (0.8 mU/l). Subject weight or injected ^18^F-FDG-activity did not differ significantly between scan times (see Table 1). CRP levels were detectable (> 1.0 mg/l) in some of the patients (4/10) during hypothyroidism, but during TSH suppression, detectable CRP levels were observed in the majority of patients (6/10), and the mean serum levels were significantly higher during TSH suppression (4.8 mg/l) than during hypothyroidism (2.9 mg/l, *p* = 0.005). In addition, blood pressure, glucose and plasma lipid levels [free fatty acids (FFA) and glycerol] decreased after levothyroxine treatment (see Table 1).

**Table 1:** Subject characteristics and laboratory results

		Hypothyroid *n* = 10	TSH suppression *n* = 10	*P* value^a^	Euthyroid *n* = 7
Age (years)		47.6 (10)**	48.2 (10)**	**0.014**	62.3 (8)
Sex (males)		2**	NA	NA	7
Weight (kg)		82.3 (15.2)	83.5 (17.3)	0.779	89.6 (5.4)
BMI (kg/m^2^)		29.2 (5.8)	29.6 (6.6)	0.508	28.5 (2.0)
Overweight (BMI > 25)		8	8	1.000	7
Obese (BMI > 30)		4	4	1.000	1
Hypercholesterolemia ^b^		0**	0**	1.000	6^b^
MAP (mmHg)		105 (20)	96 (16)	**0.025**	108 (7)
Hypertension (> 140/90 mmHg)		3*	3*	1.000	6
Smoker		1	1	1.000	0
Injected ^18^F-FDG activity		72.3 (4.1)	72.8 (4.3)	0.475	75.1 (3.3)
Levothyroxine dose (μg/day)		NA	143.8 (23.8)	NA	NA
TSH (mU/l)		104.9 (53.6)**	0.5 (0.6)**	**0.005**	2.0 (0.7)
fT4 (pmol/l)		3.4 (0.8)**	23.1 (3.9)**	**0.005**	14.9 (1.4)
CRP (mg/l)^c^		2.9 (4.5)	4.8 (5.9)**	**0.005**	2.64 (1.2)
Glucose (mmol/l)		5.0 (0.6)	5.3 (0.4)	**0.028**	5.6 (0.5)
Free fatty acids (μmol/l)		734 (166)**	562 (172)*	**0.047**	372 (152)
Total glycerol (μmol/l)		1554 (291)	967 (486)*	**0.007**	1615 (525)
Blood pool activity	SUV_mean_ JV	1.4 (0.2)	1.3 (0.3)*	**0.026**	1.4 (0.2)
	SUV_mean_ SCV	1.6 (0.3)	1.3 (0.2)*	**0.011**	1.6 (0.3)

Bold font indicates a statistically significant difference between hypothyroidism and TSH-suppression.

When applicable, means are given with the standard deviation between brackets, or the absolute number of subjects. None of the thyroid cancer patients had a history of hypercholesterolemia and none used cholesterol-lowering drugs. However, cholesterol levels were only available for *n* = 2.

Asterisks indicate significant differences compared to euthyroid patients

*NA* not applicable

*p < 0.05

**p < 0.01

^a^*P* values were calculated for differences between hypothyroidism and TSH suppression

^b^Hypercholesterolemia was defined as a total cholesterol > 5.0 mmol/l, or LDL > 3.0 mmol/l. Cholesterol values were available for *n* = 6 of the euthyroid group

^c^seven pairs were available for analysis

Blood pool activity was significantly lower after levothyroxine treatment in comparison to hypothyroidism in both the jugular vein as well as the superior cava vein (_mean_SUV_mean_ jugular vein 1.4 ± 0.2 vs 1.3 ± 0.3, *p* = 0.026 and mean SUV_mean_ superior cava vein 1.6 ± 0.3 vs 1.3 ± 0.2, *p* = 0.011).

Since the DTC patients were only scanned during hypothyroidism and TSH suppression, and thus not in the euthyroid state, the results of this study are insufficient to confirm or dismiss a U-shaped correlation between thyroid hormone levels and arterial inflammation. Therefore, we additionally screened our institutional data for euthyroid subjects who underwent a similar scan protocol. Seven euthyroid subjects were included with a mean TSH 2.0 ± 0.7 mU/l and a mean fT4 14.9 ± 1.4 pmol/l. Characteristics of these subjects can be found in Table 1. Most importantly, euthyroid subjects had more cardiovascular risk factors than the DTC patients. They were all obese males with impaired insulin sensitivity. Their average age was older than that of the thyroid cancer patients (62.3 ± 8 years versus 47.6 ± 10 years during the first scan, *p* = 0.005) and more of them had hypertension (86% vs 30%, *p* = 0.024) and/or hypercholesterolemia (100% vs 0%, *p* < 0.001). Blood pool activity in these euthyroid patients was similar to that of the DTC patients during hypothyroidism.

### Image quality

In all scans (*n* = 20 DTC and *n* = 7 euthyroid subjects), image quality was sufficient to include all aortic regions in the analysis. However, ^18^F-FDG uptake in both carotids could not be analysed in scans of one DTC and in two euthyroid participants due to motion artefacts and/or spill-in. In two other participants (one DTC and one euthyroid), the ROIs of only one carotid artery were considered representable. For all other subjects, only the carotid with the highest TBR was included for analysis. Results for the carotid arteries are therefore based on *n* = 9 DTC patients and *n* = 5 euthyroid subjects. In one lean DTC patient, VAT could not be analysed.

### Arterial TBRs in DTC patients

The SUVs for ^18^F-FDG uptake are given in supplementary table 1. The TBR_max_ per vascular territory can be found in Table 2. The _mean_TBR_max_ for all aortic territories taken together increased from 1.6 ± 0.2 to 1.8 ± 0.2 (*p* = 0.058) after levothyroxine treatment. Similar results were also found for all individual arterial territories, with the exception of the carotids, in which no difference between ^18^F-FDG uptake was found between hypothyroid state and TSH suppression. In the aortic arch and thoracic descending aorta, the increase in _mean_TBR_max_ during TSH suppression reached statistical significance (*p* = 0.031 and *p* = 0.021 respectively). In one patient, fT4 and TSH-values were both within the normal range at the time of the second scan (TSH 0.8 mU/l; fT4 14.2 pmol/l). Exploratory exclusion of this patient increased statistical significance for all TBR analyses.

**Table 2:** TBR_max_ per vascular territory

	Hypothyroidism	TSH suppression	*P* value	Euthyroid
Carotids^a^	1.7 (0.2)*	1.7 (0.3)*	0.943	1.3 (0.2)
Ascending aorta	1.7 (0.2)	1.8 (0.2)	0.340	1.7 (0.2)
Aortic arch	1.7 (0.2)	1.9 (0.2)*	**0.031**	1.5 (0.3)
Thoracic aorta	1.6 (0.2)	1.9 (0.2)*	**0.021**	1.7 (0.2)
Abdominal aorta	1.4 (0.2)	1.6 (0.2)	0.105	1.6 (0.2)
All vessels	1.6 (0.2)	1.8 (0.2)*	0.058	1.6 (0.2)

Bold font indicates a statistically significant difference between hypothyroidism and TSH-suppression.

Whole vessel (territory) analysis for TBR_max_ is given, depicted as the mean with the standard deviation between brackets. For hypothyroidism these values are based on *n* = 9, for euthyroid *n* = 5 and for TSH suppression *n* = 10. For all other vessel territories, the mean TBR_max_ is given based on *n* = 7 for euthyroid state and *n* = 10 for hypothyroidism and TSH-suppressed state

* indicates a significant difference with euthyroid patients of *p* < 0.05

^a^the highest value of both carotids was selected for further analysis

### Arterial TBRs in a control group of euthyroid subjects

**Fig. 1:**
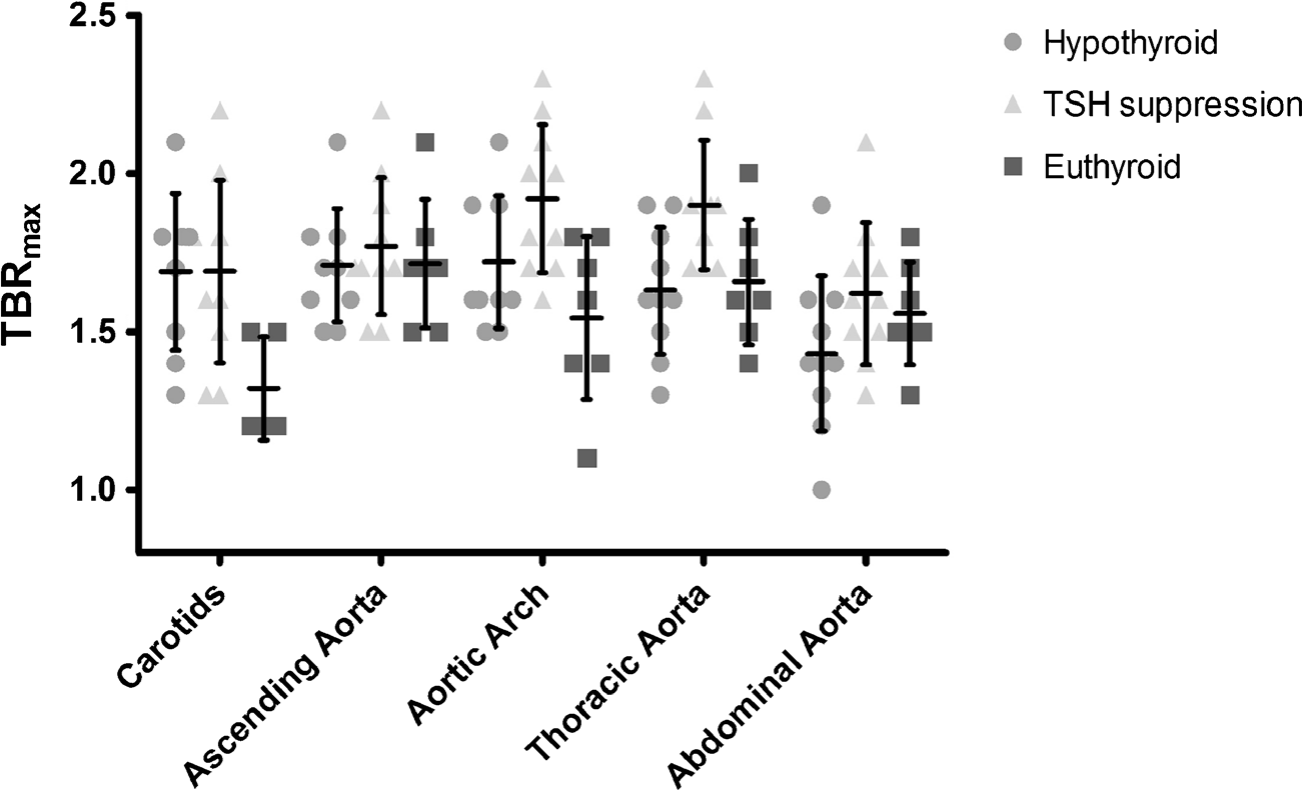
Dot plot of TBR_max_ per vascular territory during different thyroid hormone states. Mean TBR_max_ values are depicted with their standard deviation superimposed on the individual data points for the DTC patients during hypothyroidism and TSH suppression, and for euthyroid controls. TBR_max_ values are based on *n* = 10 for both hypothyroidism and TSH suppression for all aortic segments, and for the carotids during TSH suppression. TBR_max_ for the carotids during hypothyroidism is based on *n* = 9. Average TBR_max_ values for euthyroid state are based on *n* = 7 for all aortic segments and *n* = 5 for the carotids

Notwithstanding the higher number of cardiovascular risk factors in the euthyroid group, arterial TBRs were always lower in this group compared to the TBRs measured during TSH suppression in the DTC population. This was statistically significant in the carotids, aortic arch, and descending thoracic aorta (See Table 2 and Fig. 1). In addition, the arterial TBRs of euthyroid subjects were also significantly lower in comparison to the hypothyroid state in the carotid arteries and for the TBR_max_ in the aortic arch, but similar in the ascending and descending thoracic aorta, and not-significantly higher in the abdominal aorta (See Table 2 and Fig. 1). When all vascular territories were combined, there was a significant difference between the TBRs of euthyroid subjects and those under TSH suppression (*p* = 0.018 for TBR_max_).

### TBRs in spleen, bone marrow, and white adipose tissue

In agreement with the pattern of ^18^F-FDG uptake in the arteries, BM also showed significantly higher TBRs during TSH suppression (_mean_TBR_max_ 2.0 ± 0.3 vs 1.7 ± 0.3, *p* = 0.014). Differences of the ^18^F-FDG uptake between the thyroid hormone states were only slight in the spleen, VAT as well as SAT at the level of the xiphoid bone, and did not reach statistical significance. However, ^18^F-FDG uptake in nuchal SAT showed a trend towards lower TBRs during TSH suppression, in contrast to the findings in the vasculature. See Table 3 for details.

**Table 3:** TBR_max_ of the spleen, bone marrow, and adipose tissue

	Hypothyroidism	TSH suppression	*P* value
Spleen	1. 7 (0.3)	1.7 (0.2)	0.644
Bone marrow	1.7 (0.3)	2.0 (0.3)	**0.014**
SAT chest	0.4 (0.2)	0.4 (0.2)	0.550
SAT neck	0.4 (0.1)	0.4 (0.1)	0.053
VAT^a^	0.4 (0.2)	0.5 (0.1)	0.943

Bold font indicates a statistically significant difference between hypothyroidism and TSH-suppression.

TBRs are given as mean and standard deviation between brackets.

^a^*n* = 9

### Correlations between TBRs and CRP

We tested for correlations between different measures of ^18^F-FDG uptake and CRP blood levels. However, only a few of the tested correlations reached statistical significance. There were no significant correlations between CRP and the arterial SUVs. CRP was only significantly correlated to the TBR_max_ of the carotids, the ascending aorta the aortic arch, and when all vessel territories were combined for the hypothyroid period. No significant correlation between CRP and any of the ^18^F-FDG uptake measures could be found during TSH suppression or in euthyroid participants. See Table 4.

**Table 4:** Correlation between TBRs and CRP

TBR_max_	CRP levels		
	Hypothyroidism	TSH suppression	Euthyroid
Carotid	0.822**	0.374	−0.148
Ascending aorta	0.721*	0.038	0.397
Aortic arch	0.708*	0.316	0.440
Thoracic aorta	0.398	−0.114	0.145
Abdominal aorta	0.299	0.549	0.093
All vessels	0.785**	0.331	0.360
Spleen	0.663	−0.256	
Bone marrow	0.138	−0.549	
VAT	−0.074	−0.151	
SAT neck	0.140	−0.134	
SAT xyphoid	−0.244	−0.327	

Depicted are the Spearman rho (ρ) coefficients

*signifies statistical significance with a *p* value < 0.05

**indicates a *p* value < 0.01

## Discussion

In this explorative study, we were able to show that vascular TBRs, as a measure for arterial inflammation, are higher under TSH-suppressive medication than during hypothyroidism, indicating a negative impact of TSH suppression on atherosclerosis. To the best of our knowledge, changes in arterial inflammation due to thyroid hormone imbalance have never been studied before in humans in vivo. The inclusion of thyroid cancer patients enabled us to study the effects of pathological thyroid hormone levels on both ends of the spectrum within the same patient. This study design controls for differences in causation of thyroid hormone imbalance, but also for the variability in the necessary levothyroxine dose for each patient to establish adequate TSH suppression, since patients served as their own control.

We chose to focus on TBRs as an outcome measure, since TBR has been proposed as the more relevant measurement for arterial inflammation as it normalizes SUV values for ^18^F-FDG blood pool activity [16]. As mentioned in the Methods section, ROIs were drawn to encompass the entire vessel wall including the lumen, as is a common and accepted procedure in analyses of vessel wall inflammation. This approach is mainly used to address the limited resolution of PET/CT and the chance of spill-in and spill-out. In arteries without a high-degree stenosis, such as probably most commonly seen in our population, blood pool activity is a major contributor to the vessel wall SUVs. As such, this will generally result in an underestimation of the activity in the vascular wall, and fluctuations in blood pool activity can greatly affect results. Correction for the blood pool activity is therefore a necessity. This is of particular relevance in the current study, since thyroid hormone has a wide range of effects on multiple tissues and amongst others increases general glucose uptake, glycolysis, gluconeogenesis and tissue blood flow [17]. These effects will likely affect ^18^F-FDG metabolism as well. For instance, through an increased general energy expenditure [14] and increased glucose transporters (GLUT) expression in different tissues, such as the muscles, liver and brain [18–20], it is likely that competition for ^18^F-FDG is increased. Consequently, the distribution of ^18^F-FDG uptake is increased throughout the body, resulting in a lower blood pool activity and lower absolute uptake in specific tissues. In addition, thyroid hormone may also affect renal function and increase blood volume. However, although ^18^F-FDG is cleared by the kidneys, increased diuresis by means of saline infusion or diuretics did not effect ^18^F-FDG blood activity in rats [21]. Additionally, ^18^F-FDG distribution was not significantly affected in patients with a disrupted renal function [22]. Therefore, we assume the lower blood pool activity during TSH suppresion to rather be an effect of an altered distribution of ^18^F-FDG throughout the body. TBR is used to correct for the distributional spread of ^18^F-FDG. SUV already corrects for injected dose, patient weight, and decay. In a way, TBR is not a more accurate representation of the absolute uptake than SUV [23], but it is, as its name suggests, a ratio between the uptake in the target, the vessel wall (including the lumen), and the blood pool activity (in a nearby vein). Even though the absolute uptake in the vessel walls may have been lower after thyroxine depletion, the relative uptake was higher. Earlier research showed TBR to better correlate to the clinical risk of cardiovascular events and to the effects of dedicated treatment [4, 24], and it is therefore recommended in vascular research [16]. Concurrently, we found CRP levels to correlate to vascular TBRs, but not to vascular SUVs during hypothyroidism.

Previous research has mainly focused on the harmful effects of hypothyroidism on atherosclerosis [25–27]. Strong evidence exists that hypothyroidism raises the risk for the onset and progression of atherosclerosis due to an increased incidence of cardiovascular risk factors such as hypertension and a disturbed lipid balance [26, 27]. In concurrence with those findings, we found both blood pressure and plasma lipid levels (FFA and glycerol) to be higher during hypothyroidism than during TSH suppression. In addition, (subclinical) hypothyroidism has also been associated with increased systemic low-grade inflammation, as measured by elevated CRP and interleukin levels, compared to euthyroid patients [28]. In agreement with this, surgically removed carotid atherosclerotic plaques of these patients had a higher macrophage content compared to those of euthyroid patients [28]. In line with those results, other studies found a possible anti-inflammatory effect of thyroid hormone [25, 29]. In vitro, thyroid hormone decreased the migration of bone-marrow-derived monocytes from hematopoietic tissues to local inflammatory processes [29]. Further suggesting an anti-inflammatory effect of thyroid hormones, additional knockout of the thyroid hormone receptor alpha in apoE knockout mice, a commonly used atherosclerosis model, increased both atherosclerotic plaque size, and plaque inflammation [25]. Our finding that CRP levels correlate to vascular TBRs during hypothyroidism seems to underline an inflammatory effect of hypothyroidism.

In contrast, the results of our study also seem to plead for a pro-inflammatory effect of increased thyroid hormone levels and/or TSH suppression. Although we were unable to gain histological proof that the increased arterial TBRs were indeed caused by an increased number of macrophages in the vascular wall or macrophage activation, the elevated levels of CRP during TSH suppression indicate a role for inflammation. During hypothyroidism, CRP levels were detectable (> 1.0 mg/l) in 40% of the patients and similar to the CRP levels in the euthyroid participants, but during TSH suppression CRP levels were detectable in in 60% of the patients and the mean serum levels were significantly higher during TSH suppression than during hypothyroidism. Previous research has shown that increased CRP levels correlate with a significantly increased risk of subsequent cardiovascular events [30] and with arterial ^18^F-FDG uptake [31, 32].

In our study population, levothyroxine treatment modified cardiovascular risk factors classically related to hypothyroidism [26, 27], suggesting an increase of these risk factors during hypothyroidism. The observed decrease in blood pressure and plasma lipids after levothyroxine treatment, combined with the increased uptake of ^18^F-FDG in the vascular wall, might suggest that a different mechanism is responsible for the inflammatory changes in the vascular wall during TSH suppression as compared to hypothyroidism. Previously published preclinical studies have also shown direct effects of thyroid hormone on vascular wall cells [33, 34]. For instance, rats with induced thyrotoxicosis showed markers for increased oxidative stress and lipid peroxidation in addition to increased arterial stiffness, due to changes in vascular smooth muscle cell and elastin and collagen content in the vessel wall [33, 34]. Furthermore, elevated markers of endothelial activation were observed in patients with hyperthyroidism due to Graves’ disease [35, 36]. The exact mechanism underlying the higher arterial uptake of ^18^F-FDG during TSH suppression remains, therefore, to be investigated.

In DTC patients, lower TSH levels have been related to an increased risk of cardiovascular mortality [13], and other clinical studies have shown thyroid hormone levels to be related to different measurements for atherosclerotic disease burden [2, 37, 38]. For instance, high fT4 levels have been related to increased arterial stiffness [2] and low TSH levels to increased carotid intima-media thickness [37]. In a large population study, Zhang et al. [38] found both low-normal fT4 and low-normal TSH levels to be related to higher coronary artery calcium (CAC) scores [38]. The authors suggest that fT4 and TSH might influence atherosclerosis via different direct and indirect mechanisms, since TSH receptors are present along with thyroid hormone receptors in vascular smooth muscle cells. Although this study only included participants with hormone levels within the normal range, these findings might indicate that extreme hormone levels on both ends of the spectrum play a role in the development of atherosclerosis. Thus, the correlation between thyroid hormone levels and vascular disease might indeed be U-shaped [2]. This suspicion was confirmed by the results of our study when we compared arterial TBRs of euthyroid patients with those of our DTC population. Arterial TBRs were generally lower in the euthyroid group compared to both hypothyroidism and TSH suppression, despite their higher cardiovascular risk profile compared to the DTC study population. This finding supports the hypothesis that both hypothyroidism and hyperthyroidism predispose to atherosclerosis.

The results of previously published ^18^F-FDG-PET studies indicate that the hematopoietic organs are involved in the systemic inflammatory response in atherosclerosis, as the ^18^F-FDG uptake in the spleen and the bone marrow showed a significant correlation with arterial inflammation [8, 9]. In accordance with these results, we also found significantly higher bone marrow TBRs during TSH suppression compared to the hypothyroid situation, suggesting increased hematopoietic activity, potentially in the context of the observed increased arterial inflammation.

Additionally, it must be pointed out that although fT4 levels were above reference values in 90% of the patients, perfect TSH suppression was not yet confirmed by TSH values in 40% of the patients. In health, TSH responds with a logarithmic increase compared to the fT4 decrease and vice versa [12]. As such, TSH is more sensitive to endogenous thyroid disease than fT4 levels. However, its response is not instant to levothyroxine treatment and it is not uncommon to await further TSH decrease while maintaining a stable levothyroxine dose. Our findings result in the expectation that with further TSH decrease and longer duration of TSH suppression, vascular inflammation would only increase further. TSH suppression in DTC patients is a long-term treatment and previous research points to negative effects on cardiac physiology and bone density [12]. Thus far, studies on a potential increased risk of cardiovascular death in DTC patients with TSH suppression have been inconclusive [12], but our findings agree with the recent more critical view on TSH suppressive therapy, pointing to a personalized approach [10, 11].

### Limitations

This was an exploratory study, and as such it is limited by a small population sample. It is possible that some of our analyses did not reach statistical significance because the study might have been underpowered. Therefore, our findings need to be confirmed in a larger prospective study.

A drawback of our study design is the possibility of a so-called period effect, since TSH suppression always followed hypothyroidism and the duration of each period differed. Because a longer time period of TSH-suppressive therapy was needed to achieve stable thyroid hormone levels, the period between the start of levothyroxine treatment and the second scan was longer than the period between thyroidectomy and the first scan. We can therefore not completely exclude the possibility that the TBRs were higher during TSH suppression because the period of TSH suppression was longer. However, our findings seem to be in contrast to earlier studies showing a decrease in vascular disease with thyroid hormone replacement therapy in (subclinical) hypothyroidism [39, 40]. This rather supports a detrimental effect of increased thyroid hormones on arterial inflammation.

Since our scan protocol was primarily aimed at imaging cold-induced BAT activity, it differs from arterial imaging guidelines on PET imaging in atherosclerosis [16, 41]. However, since all patients served as their own control, this should not have biased our results to a relevant degree.

The inclusion of diabetes-prone participants as euthyroid controls should be considered as merely exploratory. As mentioned before, these subjects cannot be considered as fully healthy controls, but neither can they be considered as atherosclerotic.

## Conclusions

To the best of our knowledge, this is the first study linking disturbed thyroid hormone levels to arterial inflammation in vivo. This explorative study shows arterial TBRs, as a measure for arterial inflammation, to be higher during TSH suppression than during hypothyroidism, and arterial TBRs to be higher during both ends of thyroid hormone imbalance than during the euthyroid state. Future prospective and well-powered studies need to confirm our results.

## Electronic supplementary material

Supplementary table 1 (DOCX 63 kb)

## Abbreviations

^18^F-FDG: ^18^F-fluorodeoxyglucose
BAT: Brown adipose tissue
BM: Bone marrow
CAC score: Coronary artery calcium score
CRP: C-reactive protein
CT: Computed tomography
DTC: Differentiated thyroid carcinoma
FFA: Free fatty acids
fT4: Free thyroxin
GLUT: Glucose transporter
JV: Jugular vein
PET: Positron emission tomography
ROI: Region of interest
SAT: Subcutaneous adipose tissue
SUV: Standardized uptake value
SCV: Superior cava vein
TBR: Target-to-background ratio
TSH: Thyroid-stimulating hormone; thyrotropin
VAT: Visceral adipose tissue
WVA: Whole vessel analysis
