# Supplementary material for: TSH suppression aggravates arterial inflammation — an 18F-FDG PET study in thyroid carcinoma patients
Source: Eur J Nucl Med Mol Imaging. 2019 Mar 11;46(7):1428–38. doi: 10.1007/s00259-019-04292-w (PMC6533218; doi:10.1007/s00259-019-04292-w)
Supplement: Supplementary file 1 — (DOCX 63 kb) [file 259_2019_4292_MOESM1_ESM.docx]

| **Supplementary table 1 Mean and maximum arterial SUVs per territory** | | | | |
| --- | --- | --- | --- | --- |
|  |  | **Hypothyroidism** | **TSH suppression** | **P-value** |
| **Carotids ^a^** | SUV_mean_ | 1.9 (0.4) | 1.6 (0.3) | 0.116 |
|  | SUV_max_ | 2.3 (0.5) | 2.1 (0.4) | 0.115 |
| **Ascending aorta** | SUV_mean_ | 2.1 (0.4) | 1.6 (0.2) | **0.013** |
|  | SUV_max_ | 2.9 (0.4) | 2.2 (0.3) | **0.011** |
| **Aortic arch** | SUV_mean_ | 1.9 (0.4) | 1.7 (0.3) | **0.035** |
|  | SUV_max_ | 2.8 (0.5) | 2.5 (0.5) | 0.183 |
| **Thoracic aorta** | SUV_mean_ | 1.9 (0.2) | 1.7 (0.3) | **0.042** |
|  | SUV_max_ | 2.6 (0.3) | 2.4 (0.3) | 0.160 |
| **Abdominal aorta** | SUV_mean_ | 1.7 (0.3) | 1.5 (0.3) | 0.284 |
|  | SUV_max_ | 2.3 (0.4) | 2.1 (0.4) | 0.234 |
| **All vessel territories** | SUV_mean_ | 1.9 (0.3) | 1.6 (0.2) | **0.036** |
|  | SUV_max_ | 2.6 (0.4) | 2.3 (0.3) | **0.042** |

Supplementary table 1. Mean values are given with the standard deviation between brackets. ^a^ the highest value of both carotids was used for further analysis.
